# A Longitudinal Study of Alterations of Hippocampal Volumes and Serum BDNF Levels in Association to Atypical Antipsychotics in a Sample of First-Episode Patients with Schizophrenia

**DOI:** 10.1371/journal.pone.0087997

**Published:** 2014-02-13

**Authors:** Emmanouil Rizos, Matilda A. Papathanasiou, Panagiota G. Michalopoulou, Efstathios Laskos, Aggeliki Mazioti, Anastasia Kastania, Konstantina Vasilopoulou, Paraskevi Nikolaidou, Dimitrios Margaritis, Charalabos Papageorgiou, Ioannis Liappas

**Affiliations:** 1 2^nd^ Department of Psychiatry, “ATTIKON” General Hospital, Medical School, National and Kapodistrian University of Athens, Athens, Greece; 2 2nd Department of Radiology, “ATTIKON” General Hospital, Medical School, National and Kapodistrian University of Athens, Athens, Greece; 3 Department of Psychosis Studies, Section on Schizophrenia, Imaging and Therapeutics, Institute of Psychiatry, King’s College London, London, United Kingdom; 4 Department of Informatics, Athens University of Economics and Business, Athens, Greece; 5 Biochemistry and Microbiology Department, Athens Psychiatric Hospital “Dromokaition”, Athens, Greece; Chiba University Center for Forensic Mental Health, Japan

## Abstract

**Background:**

Schizophrenia is associated with structural and functional abnormalities of the hippocampus, which have been suggested to play an important role in the formation and emergence of schizophrenia syndrome. Patients with schizophrenia exhibit significant bilateral hippocampal volume reduction and progressive hippocampal volume decrease in first-episode patients with schizophrenia has been shown in many neuroimaging studies. Dysfunction of the neurotrophic system has been implicated in the pathophysiology of schizophrenia. The initiation of antipsychotic medication alters the levels of serum Brain Derived Neurotrophic Factor (BDNF) levels. However it is unclear whether treatment with antipsychotics is associated with alterations of hippocampal volume and BDNF levels.

**Methods:**

In the present longitudinal study we investigated the association between serum BDNF levels and hippocampal volumes in a sample of fourteen first-episode drug-naïve patients with schizophrenia (FEP). MRI scans, BDNF and clinical measurements were performed twice: at baseline before the initiation of antipsychotic treatment and 8 months later, while the patients were receiving monotherapy with second generation antipsychotics (SGAs).

**Results:**

We found that left hippocampal volume was decreased (corrected left HV [t = 2.977, df = 13, p = .011] at follow-up; We also found that the higher the BDNF levels change the higher were the differences of corrected left hippocampus after 8 months of treatment with atypical antipsychotics (Pearson r = 0.597, p = 0.024).

**Conclusions:**

The association of BDNF with hippocampal volume alterations in schizophrenia merits further investigation and replication in larger longitudinal studies.

## Introduction

Preclinical and clinical studies suggest a role of brain-derived neurotrophic factor (BDNF) in neuronal survival, differentiation, synaptogenesis and maintenance [1], [2]. Dysfunction of the neurotrophic system has been implicated in the pathophysiology of schizophrenia**.** Low serum BDNF levels have been linked to the onset of schizophrenic process and the duration of untreated psychosis, probably reflecting an association between BDNF and the pathogenesis of the disorder [3]–[6].

BDNF alterations in medicated patients with schizophrenia may also reflect the effects of antipsychotic treatment and possible differential impact of first generation antipsychotics (FGAs) relative to second generation antipsychotics (SGAs) [7]. For example, it has been shown that chronic patients with schizophrenia on clozapine had marginally significant higher BDNF levels compared to patients on FGAs [8], [9]. The type of atypical antipsychotic may also have differential effects on BDNF levels, as it has been shown that patients with schizophrenia on clozapine had higher BDNF levels compared to patients on risperidone [10]. However, other studies have found no effects of antipsychotic treatment on BDNF levels [11]–[13]. In addition to antipsychotic treatment, other factors such as stage of illness, gender and genetic makeup seem to play a role in BDNF levels of patients with schizophrenia [14]–[16].

Schizophrenia is associated with structural and functional abnormalities of the hippocampus, which have been suggested to play an important role in the formation and emergence of schizophrenia syndrome [17]–[20]. In terms of structural abnormalities, several magnetic resonance imaging studies and meta-analyses of the relevant studies have shown significant bilateral hippocampal volume reduction [21]–[23]. BDNF is highly expressed in the hippocampus and is associated with neuronal activation and remodeling of this brain region [2], [24], [25].

Several preclinical studies have shown that hippocampal BDNF expression is modulated after treatment with antipsychotics, with atypical antipsychotics inducing differential BDNF changes compared to typical antipsychotics. In schizophrenia, many studies have shown progressive hippocampal volume loss [23], [26]–[28]. However, the mechanisms underlying this progressive brain change, the role of antipsychotics and their association with the alterations of the neurotrophine system and BDNF in particular remain unclear [29]–[33].

In the present longitudinal study we investigated the alterations of hippocampal volume and serum BDNF levels in a sample of first episode patients with schizophrenia before the initiation and following a short term exposure to SGAs. We tested the hypothesis that alteration of serum BDNF levels following the initiation of SGAs, would be associated with alteration of HVs.

## Materials and Methods

### Subjects

Twenty drug-naive patients in their first-episode of schizophrenia (n = 20, M/F: 8/12), with a mean age 30.75±10.52 years, were recruited from the Psychiatric Department of “ATTIKON” General Hospital from November 2010 through March 2012 (Table 1). Blood samples were collected at the time of patient admission and eight months later. The first MRI scan was carried out within the first 7 days of the patients’ admission before the initiation of antipsychotic treatment. The second MRI scan was carried out 8 months after the 1^st^ brain scan.

**Table 1:** Demographic, clinical and neuroimaging characteristics of the study participants.

Patients (n = 14)	Before	After 8 months
Age (years)	29.71±10.21	
Sex (M/F)	8/6	
Marital status (M/U)	4/10	
Education (years)	13.00±3.65	
Professional status (E/U)	11/3	
PANSS positive score	30.43±3.85	19.71±3.40
PANSS negative score	28.07±8.28	18.64±6.41
DUP (months)	8.00±4.60	
Ser BDNF (ng/ml)	9.92±3.96	13.23±7.77
Whole brain volume	1295.21±246.97	1225.68±229.84
Corrected left hippocampus	2446.74±460.43	2270.70±453.62
Corrected right hippocampus	2423.28±402.25	2323.18±409.61

Marital status (married/unmarried), professional status (employed/unemployed), PANSS = Positive and Negative Syndrome Scale, DUP = duration of untreated psychosis, Ser BDNF = serum brain derived neurotrophic factor, HV = hippocampal volume.

Study participants were assessed with SCID-IV, Patient edition and Positive and Negative Syndrome Scale (PANSS) and they were followed-up monthly. Both assessments were made by an experienced Clinical Psychiatrist (ENR). Additional information was collected from the patients and also from family members or carers, where these sources where available. Patients were excluded if they had a history of any neurological disease or any other physical disease and current substance abuse or dependence in the preceding 6 months as defined by DSM-IV (APA, 2000). Exclusion criteria for the patients included deterioration in their clinical state, which would require changes in the antipsychotic treatment, regarding the type of the antipsychotic agent.

Following the first scanning session, the dose and the type of antipsychotic treatment were decided by the attending psychiatrist. Two patients were excluded because they were diagnosed –based on SCID- with brief psychotic episode. One patient withdrew consent from the study while another was excluded from the study, because of alcohol abuse during the follow-up period. The patients were followed-up monthly. During follow-up, one patient was excluded because he was diagnosed with substance abuse, two more patients were excluded because they diagnosed as brief psychotic episodes and another one dropped-out the study. Six months after the initial assessment, the DSM-IV criteria (APA 2000) were applied and all fourteen patients included in the final analysis study were diagnosed with schizophrenia (10 of paranoid subtype and 4 of disorganized subtype)**.** All patients were treated with SGAs: 4 patients were treated with olanzapine, 4 with quetiapine, 2 with risperidone, 2 with aripiprazole and 2 with amisulpride [34] (Table 2). In cases of psychomotor agitation lorazepam was administered in a few patients. Serum BDNF levels were measured in the beginning of the study prior to the initiation of antipsychotic treatment and eight months later.

**Table 2:** All patients were treated with SGAs.

Drug name	Mean	N	Std. Deviation
OLANZAPINE	375.00	4	50.00
RISPERIDONE	375.00	2	106.06
ARIPIPRAZOLE	400.00	2	0.000
AMISULPRIDE	600.00	2	282.84
QUETIAPINE	800.00	4	0.00

The study was approved by the ethics committee of University General Hospital “ATTIKON”. Written, informed consent was obtained from all research participants. Participation was voluntary and participants were allowed to withdraw at any point with no disadvantage to their treatments. All participants were examined by an expert clinical psychiatrist to certify that their capacity to consent was not compromised by their mental status. Furthermore family relatives or care takers were present to assure that the participants fully understood before giving their consent.

### Methods

### 

#### MRI procedure & BDNF measurement

MRI examinations of the brain on all patients and control subjects were performed using a Philips Intera 1.5T system with a quadrature head coil and included routine dual echo axial images (proton density and T2) and sagittal T1 sequence. Volumetric studies were performed using a T1-weighted 3D/FFE gradient-echo sequence with the following parameters TR/TE 14.3/3.3 msec, flip angle 30°, field of view 240, matrix 256×256, slab thickness 3 mm overcontiguous with 1.5 mm spacing and 124–130 partitions with an inplane resolution of 0.94×0.94 mm. All data acquisition was performed in the coronal plane, which was perpendicular to the anterior commissure-posterior commissure (AC-PC) line. The whole brain was covered. Each acquisition was transferred to a View Forum work station (Philips Medical Systems) and analyzed using software (Philips Medical Systems Release 4.1,V1L2). Analysis was performed after tracing the boundaries of the hippocampus manually to create a closed contour, on each section. The area of each enclosed region was estimated by pixel based volumetry. The areas of all sections were added and the sum was multiplied by 1.5 (the section thickness) to determine individual volume for each hippocampus. Both the right and the left hippocampi were measured. The landmarks used for identification of the hippocampus were those described by Cook et al [35]. Intrarater variability was 0,967 for hippocampal volume (HV) measurements. Total cerebral volume was measured on coronal sections by manually tracing the cerebral hemispheres along the outer brain surface excluding the cerebellum and brain stem. For total cerebral volume measurements were performed on one in every ten consecutive sections equally spaced through the cerebrum. Intrarater variability was 0.999 for total intracerebral volume measurements. HVs were normalized to total cerebral volume according to a previously described method [36]. Corrected hippocampal volume was used for our analyses. Corrected hippocampal volume refers total brain volume as described elsewhere [36] and as indicated below. In this study corrections of hippocampal volume have been applied by the covariance method, as described by Jack et al. This method derives a corrected hippocampal value via the following equation: NV = OV-Grad (CM1-CM mean) where NV is corrected hippocampal volume, OV is original hippocampal volume, Grad is the gradient of the regression line between the hippocampal volume and the cerebral measure, CM1 is the value of the appropriate cerebral measurement for that subject, and CM mean is the mean value of that measure for all control subjects.

Regarding the *p*reparation of serum and storage, human sera were obtained by drawing blood in serum collection Vacutainer tubes (Becton-Dickinson, Rutherford, NJ). The samples were allowed to clot for 30 minutes before centrifuged at 3500 rpm for 15 min at 15°C. Serum was carefully separated and stored at –20°C until analyzed.

Concerning Measurement of BDNF levels, serum BDNF levels were quantitated in the rethawed serum samples by Quantikine Immunoassay Kit (Catalog No. DBD000) of R&D Systems (Minneapolis, MN 55413, USA). This was a double antibody sandwich ELISA method. The manufacturer instructions were applied to develop the kit, to the calibration method and to the measurement of the samples. The absorbance was measured at 450 nm and corrected at 570 nm by Mediators PhL microplate reader (Mediator Diagnostika Gmbh, Vienna, Austria). The minimum detectable dose of BDNF was typically less than 20 pg/ml (0.02 ng/nl).

#### Statistical analyses

To ensure the stability and the reliability of our findings we performed statistical analysis using Bootstrapping to produce accurate and reliable results. We have used IBM SPSS Bootstrapping which estimates the sampling distribution of an estimator by re-sampling with replacement from the original sample. Through re-sampling, SPSS Bootstrapping can create thousands of alternate versions of a dataset, providing a more accurate view of what is likely to exist in the population. (Its default setting is 1.000 samples). The Reliability analysis was performed in this study using the Cronbach alpha algorithm.

The differences in corrected left hippocampus were calculated subtracting the value of corrected left hippocampus before the drug initiation from the value of corrected left hippocampus following the drug initiation. Similar calculation logic was used to derive the differences in corrected right hippocampus. The normality of the distributions for age, BDNF, corrected left hippocampus and corrected right hippocampus, differences in corrected left hippocampus, differences in corrected right hippocampus, BDNF level change, PANSS positive and PANSS negative scores was tested using the Kolmogorov–Smirnov Test. We used paired t-test with Bootstrap for the comparison of serum BDNF, corrected left hippocampus and corrected right hippocampus, PANSS positive scores as well as PANSS negative scores, before and after the drug initiation. To investigate the association between BDNF levels and hippocampus volume, we performed multiple regression through the origin with Bootstrap (Sampling method: simple. Number of samples: 1000. Confidence Interval Level: 95.0%. Confidence Interval type: Bias-corrected and accelerated (BCa). Number of samples: 1000). The corrected left hippocampus at the endpoint of the study (i.e. 8 months after the initiation of antipsychotic treatment) was defined as the dependent variable and the corrected left hippocampus at study entry and BDNF level at study endpoint were defined as the predictors. We have also investigated the possibility to build a prediction model using as dependent variable the corrected right hippocampus at the endpoint of the study and as predictors corrected right hippocampus at study entry and BDNF level at study endpoint using multiple regression through the origin with Bootstrap. We have also used Pearson Correlation analysis with Bootstrap to examine the linear relationships between the differences in corrected left hippocampus and BDNF level change as well as the differences in corrected right hippocampus and BDNF level change. The statistical significance level was defined at p<0.05 (2-tailed).[37]–[39].

## Results

**Figure 1:**
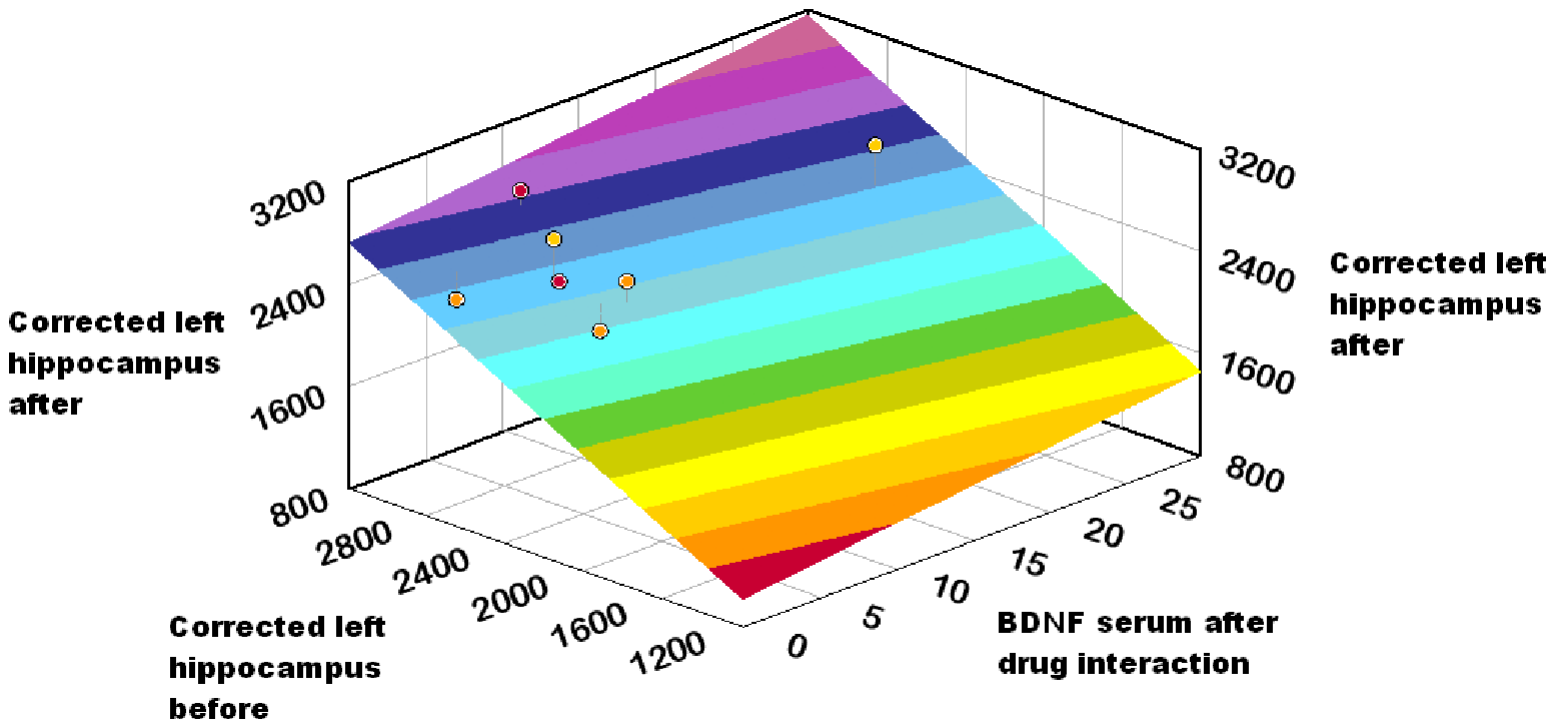
Model fit of corrected left hippocampus at the study endpoint (as dependent variable). Predictors: serum BDNF after drug interaction, Corrected left hippocampus before.

**Figure 2:**
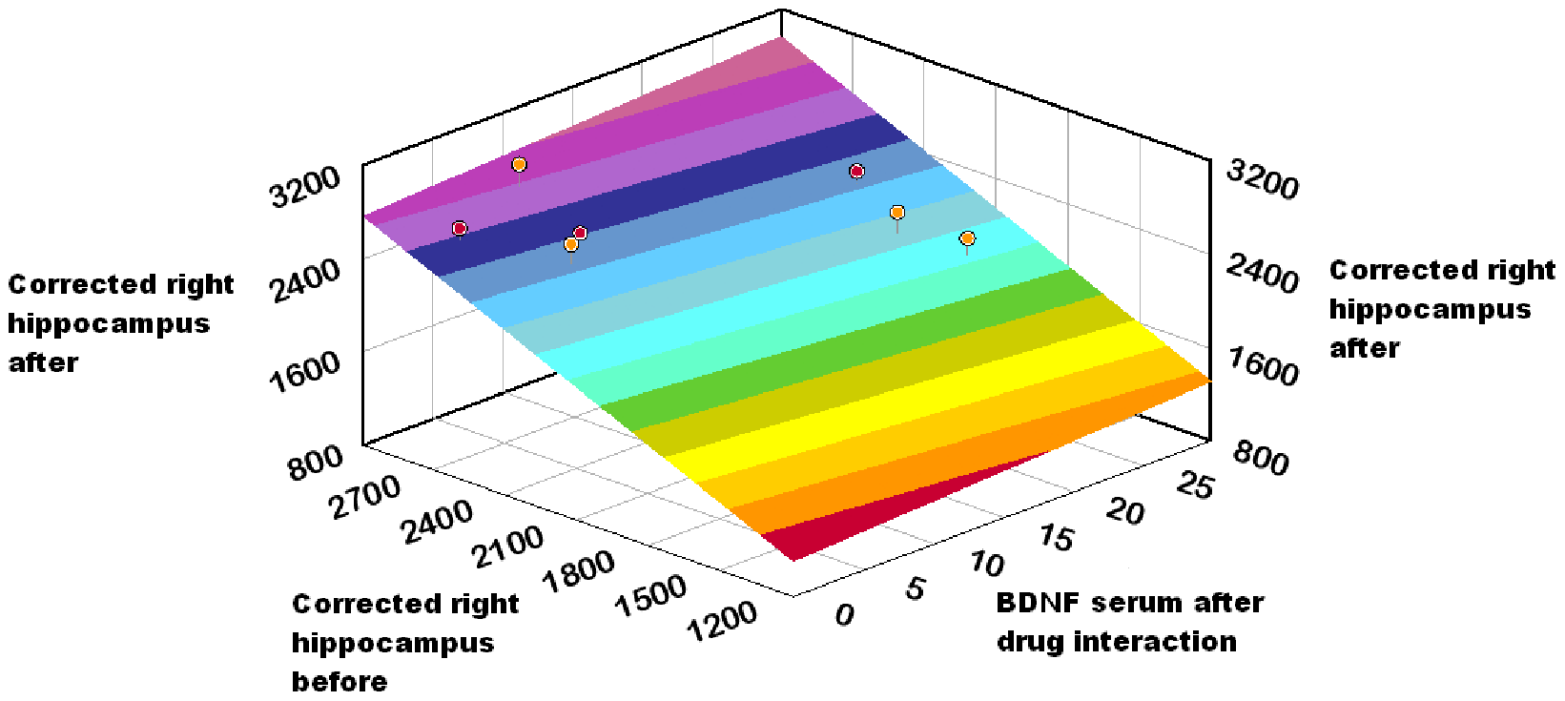
Model fit of corrected right hippocampus at the study endpoint (as dependent variable). Predictors: serum BDNF after drug interaction, Corrected right hippocampus before.

**Figure 3:**
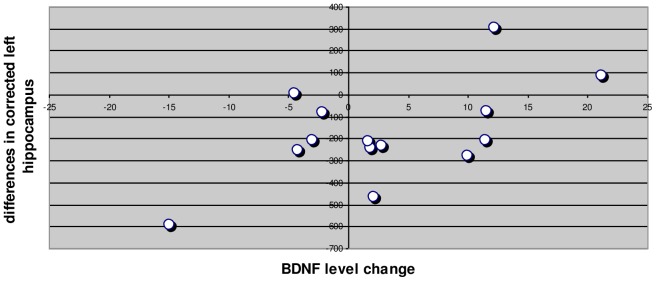
Differences in corrected left hippocampus and BDNF level change. Pearson r = 0.597, p = 0.024. Bootstrap: Sampling method single- Bias = −0.37, Std. Error = 0.214, 95% Confidence interval 0.021 to 0.863.

**Figure 4:**
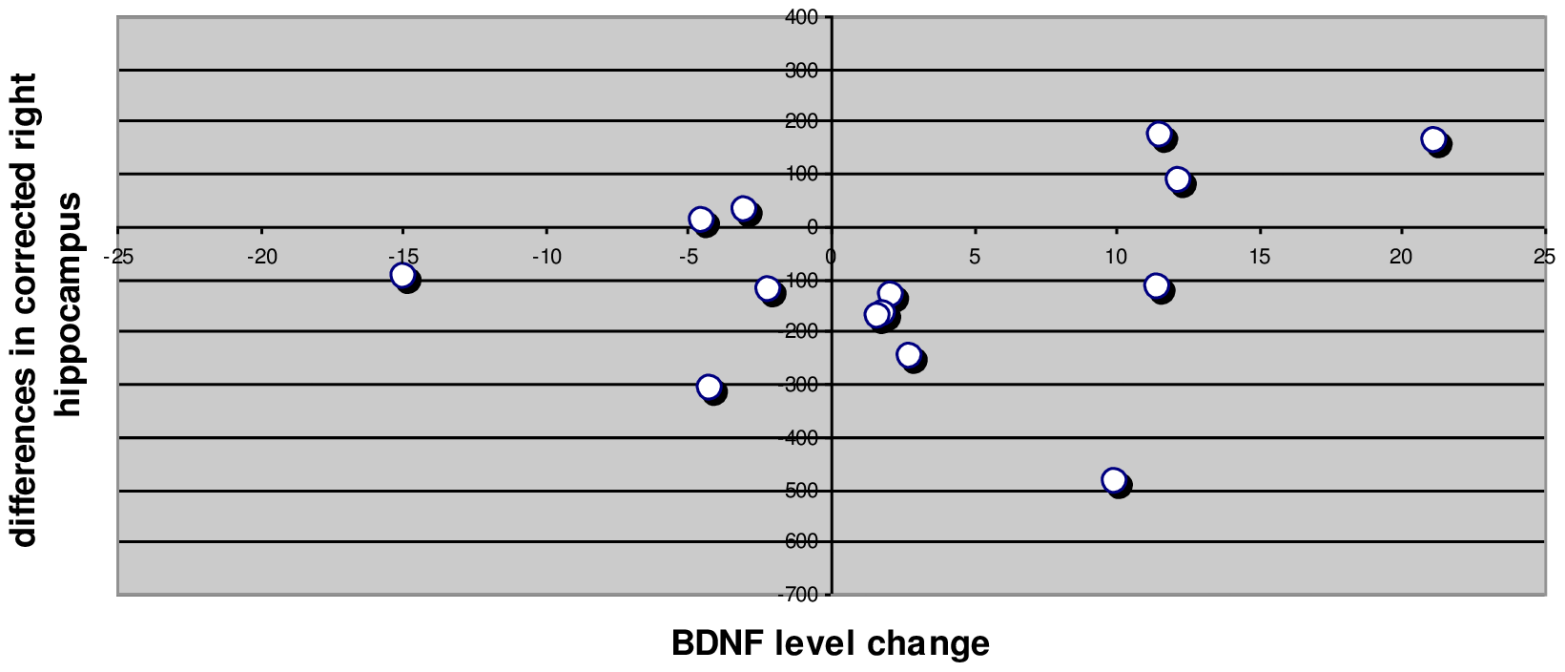
Differences in corrected right hippocampus and BDNF level change. Pearson r = 0.281, p = 0.330 (NS). Bootstrap Sampling method single. Bias = 0.010, Std. Error = 0.293, 95% Confidence interval −0.409 to 0.752.

To ensure the reliability of the sample, we successfully tested it against the Cronbach alpha algorithm (a = *0.748*). Using paired t-test, statistically significant differences between the mean values of PANSS positive subscale scores (t = 12.093, df = 13, p = .000) and PANSS negative subscale scores (t = 7.139, df = 13, p = .000) before and after the initiation of antipsychotic medication were revealed. Significant differences were found between corrected left hippocampus (t = 2.977, df = 13, p = .011) and marginally between corrected right hippocampus (t = 2.059, df = 13, p = .060) before and after the initiation of the antipsychotic medication. No significant differences were found in the serum BDNF levels before and after the initiation of antipsychotic medication (t = −1.343, df = 13, p = 0.202). Paired t-test with Bootstrap confirms these findings (Table 3). The regression model through the origin of the corrected left hippocampus at the study endpoint (as dependent variable) with predictors serum BDNF after drug interaction, corrected left hippocampus before produced an R square R Square = 0.995, which was significant (F = 1167.51, p = 0.000). The corrected left hippocampus at study entry was positively related to the corrected left hippocampus at the study endpoint entry (B = 0.915, t = 24.55, p = 0.000), as with BDNF level at study endpoint (B = 0.097, t = 2.6, p = 0.023) (Tables 4,5 & Figure 1). The regression model through the origin of the corrected right hippocampus at the study endpoint (as dependent variable) with predictors serum BDNF after drug interaction, corrected right hippocampus before produced a non-significant regression coefficient for BDNF level at study endpoint (B = 0.044, t = 1.189, p = 0.258) (Tables 6,7 & Figure 2). Pearson correlation analysis with bootstrap revealed the linear relationship (Pearson r = 0.597, p = 0.024) in the differences in corrected left hippocampus and BDNF level change (Figure 3). Investigation for the relationship of the differences in corrected right hippocampus and BDNF level change resulted to a Pearson r = 0.281, p = 0.330 (non significant) (Figure 4).

**Table 3:** Bootstrap for paired samples t-test.

Bootstrap for Paired Samples Test						
		Bootstrap^a^				
	Mean	Bias	Std. Error	Sig. (2-tailed)	BCa 95% Confidence Interval	
					Lower	Upper
serum BDNF before drug interaction –serum BDNF after drug interaction	−3.307	−0.0439	2.4285	0.198	−8.202	1.931
panspos1 - panspos2	10.714	0.037	0.859	0.001	9.214	12.440
pansneg1 - pansneg2	9.429	−0.022	1.267	0.004	7.429	11.929
Corrected left hippocampus before –Corrected left hippocampus after	176.043	−1.838	57.901	0.005	61.082	272.979
Corrected right hippocampus before –Corrected right hippocampus after	100.108	−0.,696	47.001	0.077	13.379	182.234

Sampling method: simple. Number of samples: 1000. Confidence Interval Level: 95.0%. Confidence Interval type: Bias-corrected and accelerated (BCa). Results are based on 1000 bootstrap samples. Comparisons between PANSPOS, PANSNEG and corrected left hippocampus are statistical significant.

a. Unless otherwise noted, bootstrap results are based on 1000 bootstrap samples.

**Table 4:** Final multiple linear regression model through the origin of corrected left hippocampus at the study endpoint (as dependent variable).

Independent variables	b	SE_b_	Beta	t statistic	p-value
corrected left hippocampusat study entry	0.851	0.035	0.915	24.548	0.000
BDNF level atstudy endpoint	14.751	5.668	0.097	2.602	0.023

Predictors: serum BDNF after drug interaction, Corrected left hippocampus before. R Square = 0.995. Regression coefficients (b), standard error of b (SE_b_), Beta and t statistic with the corresponding p-value.

**Table 5:** Bootstrap for coefficients.

Independent variables			Bootstrap				
		B	Bias	Std. Error	Sig. (2-tailed)	BCa 95% Confidence Interval	
						Lower	Upper
Corrected left hippocampus at study entry BDNF level		0.851	0.002	0.037	0.001	0.783	0.937
at study endpoint		14.751	−0.388	6.331	0.040	1.069	26.012

Sampling method: simple. Number of samples: 1000. Confidence Interval Level: 95.0%. Confidence Interval type: Bias-corrected and accelerated (BCa). Results are based on 1000 bootstrap samples. Final multiple linear regression model through the origin of corrected left hippocampus at the study endpoint (as dependent variable); Predictors: serum BDNF after drug interaction, Corrected left hippocampus before. R Square = 0.995.

**Table 6:** Final multiple linear regression model through the origin of corrected right hippocampus at the study endpoint (as dependent variable).

Independent variables	b	SE_b_	Beta	t statistic	p-value
corrected right hippocampus at study entry	0.922	0.035	0.961	26.065	0.000
BDNF level at study endpoint	6.786	5.710	0.044	1.189	0.258 (NS)

Predictors: serum BDNF after drug interaction, Corrected right hippocampus before. R Square = 0.995. Regression coefficients (b), standard error of b (SE_b_), Beta and t statistic with the corresponding p-value. NS : non significant.

**Table 7:** Bootstrap for coefficients.

Independent variables		Bootstrap				
	B	Bias	Std. Error	Sig. (2-tailed)	BCa 95% Confidence Interval	
					Lower	Upper
corrected right hippocampusat study entry	0.922	0.002	0.031	0.001	0.850	1.008
BDNF level at study endpoint	6.786	−0.687	6.458	0.255	−9.786	14.277

Sampling method: simple. Number of samples: 1000. Confidence Interval Level: 95.0%. Confidence Interval type: Bias-corrected and accelerated (BCa). Results are based on 1000 bootstrap samples. Final multiple linear regression model through the origin of corrected right hippocampus at the study endpoint (as dependent variable); Predictors: serum BDNF after drug interaction, Corrected right hippocampus before. R Square = 0.995.

## Discussion

In the present longitudinal study we found that in a sample of FEP drug-naïve patients with schizophrenia, the volume of left hippocampus was reduced 8 months after the initiation of antipsychotic medication. We also found that the BDNF levels change after 8 months of treatment with atypical antipsychotics had a significant positive contribution in the differences of corrected left hippocampus, such that the higher the BDNF levels change the higher the differences of corrected left hippocampus after 8 months of treatment with atypical antipsychotics.

Our results regarding the hippocampal volume loss are in agreement with the findings of a systematic review and meta-analysis of the magnetic resonance imaging studies in FEP patients, which identified hippocampus among the brain regions that show progressive volume loss over time in longitudinal studies [27], [29]. It has been hypothesized that brain tissue loss is more pronounced in the initial stages of the schizophrenia [32], [40]. Offering support to this hypothesis, a meta-analysis of the longitudinal neuroimaging studies in schizophrenia, found that the effect sizes of brain tissue loss in FEP patients were larger compared to the whole sample of the patients included in the meta-analysis [41].

The role of antipsychotic medications in the volume alterations of brain structures, and hippocampus in particular, in schizophrenia and the contribution of BDNF in this process remain unclear [6], [42]–[45]. It is known that BDNF is implicated in neuronal proliferation and survival, axonal and dendritic growth and remodeling assembly and remodeling of the cytoskeleton, membrane trafficking and fusion and synapse formation, function and plasticity. Many preclinical studies have investigated the role of BDNF in the mechanism of action of antipsychotics and have raised the question as to whether antipsychotics, and in particular SGAs, exert neuroprotective effects through modulation of BDNF levels [7], [46], [47], which could in turn protect against brain tissue loss [48]. Interestingly, a recent meta-analysis of the longitudinal magnetic resonance imaging studies in schizophrenia found that treatment with atypical antipsychotics reduces the progressive loss of cortical gray matter in the brain, especially in the temporal lobe [41]. A possible mechanism of this action could be through the neuroprotective effects of BDNF [1], [41]. A recent large longitudinal neuroimaging study showed that higher exposure to atypical antipsychotic medication was related to a smaller decrease in hippocampal volume over time and these findings have been suggested to reflect neuroprotective effects of SGAs on hippocampal volume [49]. Conversely, a longitudinal neuroimaging study in first-episode patients with schizophrenia showed that 6 months after the initiation of a SGA, quetiapine, patients had significant hippocampal volume loss, which was more pronounced with higher doses of quetiapine [28]. Our study sample was not large enough to study the differential effects of the different atypical antipsychotics on hippocampal volume. The specific role of different type of antipsychotics in changes of hippocampal volume is still unclear. Treatment with olanzapine and risperidone has been associated with larger hippocampal volumes in patients with schizophrenia, compared to patients treated with haloperidol in a cross-sectional study [50]. Conversely, two longitudinal neuroimaging studies found no relationship between type of antipsychotic medication and hippocampal volume change [51], [52].

We also found that the higher the BDNF levels change, the higher were the differences of corrected left hippocampus after 8 months of treatment with atypical antipsychotics. The association between serum BDNF levels and antipsychotics remains poorly understood and relevant studies have given contradictory results: while some studies have indicated the different role of some SGAs (clozapine, quetiapine or risperidone) in serum BDNF levels compared to FGAs [8]–[11], [15], [53]–[56], some others failed to find any differences in serum BDNF levels following treatment with antipsychotics [16], [57], or even any differences in serum BDNF levels between patients with schizophrenia and healthy controls [58], [59]. Since our study population consisted of drug naïve FEP patients treated with SGAs, factors that could have contributed to the lack of BDNF levels alterations following antipsychotic treatment initiation may include, body mass index, smoking status, physical activity and cognitive function [54], [60], [61].

Among the limitations of our study we should acknowledge the small sample size. However, drug-naïve first-episode patients with schizophrenia are difficult to ascertain and follow-up. BDNF levels were assessed in serum, thus representing an indirect measurement of brain BDNF levels. However, several studies have reported positive correlations between serum BDNF levels and BDNF in brain regions such as the hippocampus [62]–[64], suggesting an association between peripheral and central sources of BDNF. Another limitation of our study is the diagnostic kit we used for the measurements of serum BDNF levels. This kit (BDNF ELISA kit – R & D System) could recognize both proBDNF (precursor BDNF) and BDNF (mature BDNF) because the selectivity of BDNF antibody [65], [66]. It is well known that proBDNF and mature BDNF have opposite effects in the CNS.

In conclusion, in this longitudinal study we found reduction of left hippocampus volume 8 months after the initiation of antipsychotic medications and a significant positive contribution of the BDNF levels on the volume of left hippocampus, such that the higher the BDNF levels the higher the volume of left hippocampus after 8 months of treatment with antipsychotics. The association of BDNF with hippocampal volume alterations in schizophrenia merits further investigation and replication in larger longitudinal studies.
